# Pigmented epithelioid melanocytoma: two cases with dermoscopic findings^؆^

**DOI:** 10.1016/j.abd.2026.501309

**Published:** 2026-03-23

**Authors:** Luísa Homem de Mello Maciel Campilongo, Julian Gonzales Fraga, Gilles Landman, Francisco Macedo Paschoal

**Affiliations:** a Department of Dermatology, Centro Universitário Faculdade de Medicina do ABC, Santo André, SP, Brazil; b Department of Pathology, Escola Paulista de Medicina, Universidade Federal de São Paulo, São Paulo, SP, Brazil

Dear Editor,

Pigmented Epithelioid Melanocytoma (PEM) is a rare melanocytic neoplasm that primarily affects young adults on the extremities, with no known association with sun exposure or ethnicity.^1^, ^2^ Clinically, PEM presents as a slowly growing blue-to-black plaque or nodule larger than 1 cm. Despite its indolent course, regional lymph node metastases occur in approximately 41% of cases.^1^ The main differential diagnoses include melanoma and blue nevus.^2^

In dermoscopy, no specific structural patterns reliably distinguish PEM from melanoma. Definitive diagnosis is rendered only by histopathology. We report two pediatric patients, highlighting the dermoscopic findings suggestive of PEM, later confirmed by biopsy.

## Case 1

**Fig. 1:**
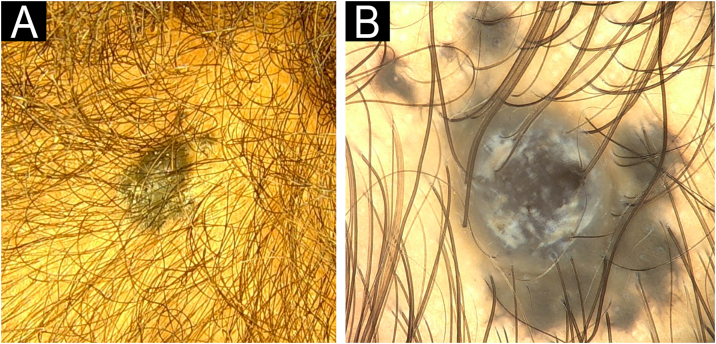
Case 1. (A) Asymmetric, poorly defined papule on the right parietal region. (B) Dermoscopy showing blue-gray pigmentation with white shiny linear streaks (20× magnification).

**Fig. 2:**
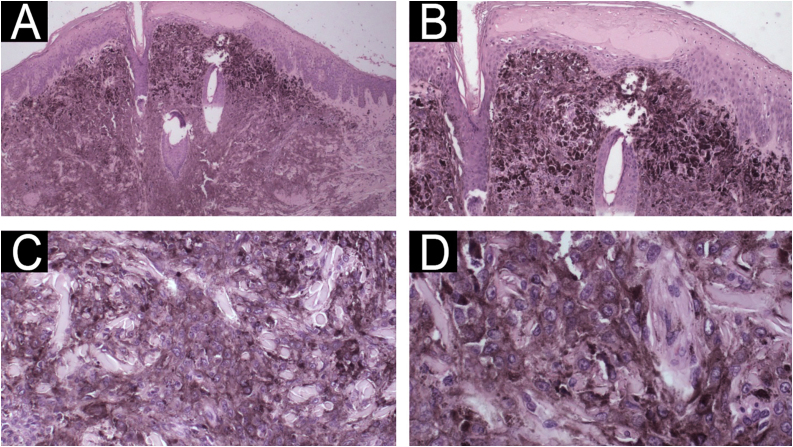
Case 1 ‒ Histopathology. (A‒B) Diffuse proliferation of neoplastic cells with abundant cytoplasm and coarse melanin pigmentation, infiltrating the full thickness of the dermis and focally extending into the hypodermis (Hematoxylin & eosin, ×40, ×100). (C‒D) The nuclei are round to oval, hyperchromatic, and exhibit prominent central nucleoli (Hematoxylin & eosin, ×200, ×400).

A 6-year-old male, Fitzpatrick IV, presented with a 1 cm growing, asymmetric, poorly defined papule on his right parietal region, evolving over four months (Fig. 1A). Dermoscopy revealed homogeneous blue-gray pigmentation, with adherent scales, and white shiny linear streaks (Fig. 1B). Differential diagnoses included melanoma, Reed nevus, and PEM. Histopathology confirmed PEM, with diffuse proliferation of neoplastic cells with abundant cytoplasm, coarse melanin pigmentation, prominent central nucleoli, and vertical growth phase (Breslow 2.5 mm) (Fig. 2). The patient awaits margin enlargement surgery in our service. No clinical changes have been observed during follow-up. This case was previously reported by de Oliveira et al.^3^ and is now presented together with an unpublished pediatric case, allowing comparison between two patients and adding new histopathological, dermoscopic, and conceptual insights into the discussion of PEM.

## Case 2

**Fig. 3:**
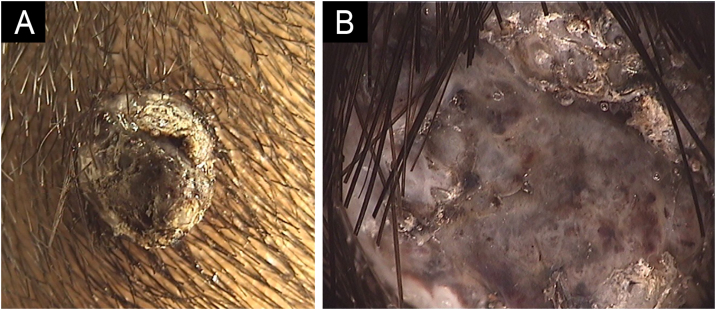
Case 2. (A) Dark, pedunculated, 1 cm tumor on the right occipital region. (B) Dermoscopy showing dark pigmentation, adherent scales, ulceration, polymorphous vessels, and white shiny streaks (20× magnification).

**Fig. 4:**
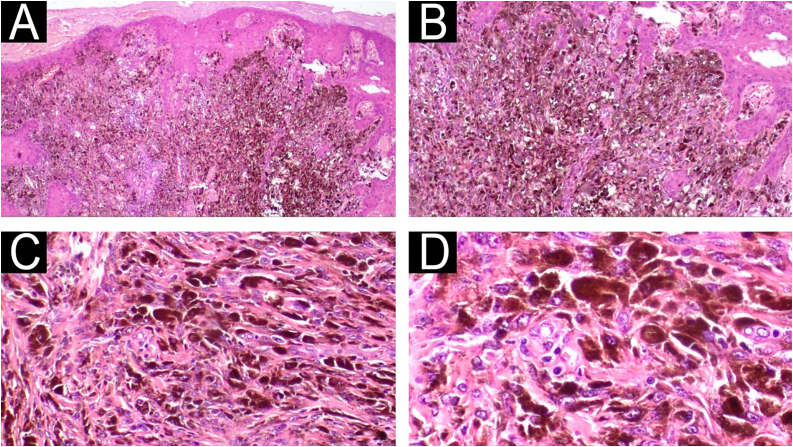
Case 2 ‒ Histopathology. (A‒B) Diffuse proliferation of epithelioid cells, some containing melanin pigmentation, infiltrating the papillary dermis in blocks and as isolated cells. A lymphocytic inflammatory infiltrate and melanophages are also noted in the dermis (Hematoxylin & eosin, ×40, ×100). (C‒D) The nuclei are hyperchromatic, polymorphic, and exhibit prominent nucleoli (Hematoxylin & eosin, ×200, ×400).

A 12-year-old male, Fitzpatrick III, presented with a 1 cm, dark, pedunculated tumor on his right occipital region evolving over five years (Fig. 3A). Dermoscopy showed dark pigmentation, adherent scales, ulceration, polymorphous vessels, and white shiny streaks (Fig. 3B). Differential diagnosis included keratoacanthoma, nodular melanoma, and pyogenic granuloma. Histopathology revealed PEM, with a diffuse proliferation of epithelioid cells, some containing melanin pigmentation, infiltrating the papillary dermis, and a vertical growth phase (Breslow 5.5 mm) (Fig. 4). Staging did not reveal metastases. Six months later, surgical margin enlargement confirmed clear margins. The patient was satisfied with the cosmetic outcome and remains under dermatologic surveillance.

PEM exhibits diverse dermoscopy features, requiring careful differentiation from other melanocytic lesions. Moscarella et al. described consistent homogeneous blue pigmentation with a range of black, white, or brown tones, while white shiny streaks, blue-white vessels may appear in advanced tumors.^4^

Histologically, PEM features a dermal proliferation of heavily pigmented epithelioid cells with abundant melanophages and infiltrative borders.^1^, ^2^, ^5^ The absence of clear epithelioid cells, nuclear pleomorphism, and the presence of dermal sclerosis weaken PEM diagnosis.^1^, ^4^, ^5^

Dermoscopy of nodular, blue-white pigmented lesions often raises suspicion for nodular melanoma, particularly when blue-white veils, pseudopods, radial streaming, or peppering structures are present. However, in the absence of these features, lesion enlargement should guide the differential diagnosis.^1^, ^4^

PEM and melanoma share abundant melanophages, ulceration, and necrosis from a histological perspective. Though atypical mitotic figures, vascular invasion, and mitotic rates are more frequent in melanoma.^1^, ^5^

Currently, PEM is the unifying term for lesions previously described as animal-type melanoma and Epithelioid Blue Nevus (EBN) of Carney complex. Clinically and histopathologically, these tumors are indistinguishable.^1^, ^2^

Molecularly, two alterations prevail: inactivation of PRKAR1A, characteristic of Carney complex, and PRKCA fusions, particularly in younger patients. Additional mutations occasionally observed in the melanocytic lineage, such as GNAQ/GNA11, link PEM to the blue nevus spectrum. The absence of TERT promoter mutations or unbalanced copy-number changes typical of melanoma reinforces its classification as an intermediate-grade melanocytic neoplasm.^1^

Although lymph node involvement is frequent (41%), systemic spread is rare (3%), and disease-specific mortality is uncommon.^1^ Management is based on wide surgical excision, and sentinel lymph node biopsy may be considered given the tumor’s uncertain malignant potential. Regular dermatologic and lymph node evaluations are recommended during follow-up.^1^, ^2^

This letter highlights a rare melanocytic neoplasm in pediatric patients, emphasizing dermoscopic, histopathologic, and molecular findings. The overlap between PEM and melanoma features makes diagnosis challenging, and the exclusion of melanoma remains the primary concern.^1^, ^3^ All suspected cases should be referred to specialized centers for accurate diagnosis and management.^1^, ^2^

## Financial support

None declared.

## Author's contribution

Luísa Homem de Mello Maciel Campilongo: Data collection, or analysis and interpretation of data; writing of the manuscript or critical review of important intellectual content; critical review of the literature; final approval of the final version of the manuscript.

Julian Gonzales Fraga: Data collection, or analysis and interpretation of data; intellectual participation in the propaedeutic and/or therapeutic conduct of the studied cases; final approval of the final version of the manuscript.

Gilles Landman: Data collection, or analysis and interpretation of data; intellectual participation in the propaedeutic and/or therapeutic conduct of the studied cases; final approval of the final version of the manuscript.

Francisco Macedo Paschoal: Study concept and design; writing of the manuscript or critical review of important intellectual content; effective participation in the research guidance; intellectual participation in the propaedeutic and/or therapeutic conduct of the studied cases; final approval of the final version of the manuscript.

## Research data availability

Does not apply.

## Conflicts of interest

None declared.

## ORCID ID

Luísa Homem de Mello Maciel Campilongo: 0000-0002-1555-807X

Julian Gonzales Fraga: 0009-0003-6019-584X

Gilles Landman: 0000-0001-7441-3068

Francisco Macedo Paschoal: 0000-0002-6264-1538
